# THE INFLUENCE OF ARTIFICIAL INTELLIGENCE ON CLINICAL DECISION MAKING: GERIATRIC REGISTERED NURSES’ PERSPECTIVES

**DOI:** 10.1093/geroni/igad104.3648

**Published:** 2023-12-21

**Authors:** Shannon Freeman, Mary Henderson-Betkus, Leanne Currie, Davina Banner, Piper Jackson

**Affiliations:** University of Northern British Columbia, Prince George, British Columbia, Canada; University of Northern British Columbia, Prince George, British Columbia, Canada; University of British Columbia, Vancouver, British Columbia, Canada; University of Northern British Columbia, Prince George, British Columbia, Canada; Thompson Rivers University, Kamloops, British Columbia, Canada

## Abstract

The emergence of ChatGPT highlights the potential of AI applications used in nursing care. AI in the form of machine learning or deep learning provides the ability to scan through clinical data using various algorithms to predict a clinical outcome for a client/patient and influencing clinical decisions. Registered nurses (RNs) are accountable for their decisions when caring for older adults receiving geriatric services and supports. Therefore, we conducted an online survey online cross-sectional survey from April to July 2023 to evaluate community focused RNs knowledge and awareness of the use of AI within their decision-making process. 233 RNs completed the survey. RNs were aware of AI in healthcare (56.7%) and in nursing (30.9%) with less familiarity with machine learning (ML) in geriatric nursing (16.3%). Source of knowledge of AI and ML came from informal sources such as media and social media and from family and friends whereas formal sources were less common. RNs agreed (69.8%) that automated outcomes to help them prioritize older adult clients and target services would be useful. However, 77.9% were concerned with who would be responsible if AI offered incorrect recommendations. Most RNs (90.2%) identified they should have the competency of how AI can improve processes and outcomes. RNs should be consulted if AI had implications on geriatric practice. Future phases of this research will examine how RNs can be better involved in AI that influences their practice and how they might be better prepared to evaluate AI outputs as part of their decision-making process.

